# Resolution of Left Ventricular Thrombus Secondary to Tachycardia-Induced Heart Failure with Rivaroxaban

**DOI:** 10.1155/2014/814524

**Published:** 2014-11-13

**Authors:** Kosuke Nakasuka, Shigenori Ito, Tsubasa Noda, Takahiro Hasuo, Satoru Sekimoto, Hiroyuki Ohmori, Masahiko Inomata, Takayuki Yoshida, Nozomu Tamai, Tomoaki Saeki, Shin Suzuki, Yoshimasa Murakami, Koichi Sato

**Affiliations:** Division of Cardiology, Nagoya City East Medical Center, 1-2-23 Wakamizu, Chikusa-ku, Nagoya-shi, Aichi 464-8547, Japan

## Abstract

A 42-year-old man was admitted to our hospital because of lumbago and tachycardia-induced heart failure. Transthoracic echocardiography revealed impaired left ventricular function and a ball mass of thrombus in the left ventricle (LV). He was found to have systemic embolism in the spleen, kidneys, brain, and limbs. The patient was treated with limb thrombectomy followed by anticoagulation. Seven days after the direct factor Xa inhibitor, rivaroxaban, was initiated, transthoracic echocardiography was repeated, revealing disappearance of the LV thrombus without any clinical signs of cardiogenic embolism. His heart failure responded well and the LV wall motion had improved. This case suggests rivaroxaban has fibrinolytic effects on thrombi even in the LV.

## 1. Introduction

Since both ischemic stroke and systemic embolism are fatal disorders, patients with atrial fibrillation (AF) are encouraged to undergo anticoagulant therapy [1]. For a long time, the vitamin K antagonist (VKA) warfarin had been used as an anticoagulant agent, but recently, novel oral anticoagulants (NOACs) have become an alternative prophylaxis for thromboembolism in patients with nonvalvular AF. NOACs have been reported to be both superior and noninferior to warfarin in preventing stroke and/or systemic embolism in patients with AF [2–5]. Some case reports have presented the resolution of left atrial appendage (LAA) thrombus with NOACs in patients with AF these days [6–8]. However, clinical experience with NOACs concerning their effects on left ventricular thrombus is still limited.

## 2. Case Presentation

An obese 42-year-old man was first seen in our hospital because of sudden onset of lumbago in association with functional NYHA class III heart failure. He had suffered from cough and dyspnea on exertion for two months and felt general fatigue and abdominal pain for a few days. His medical history included irritable bowel syndrome, but he was not taking any medication.

**Figure 1:**
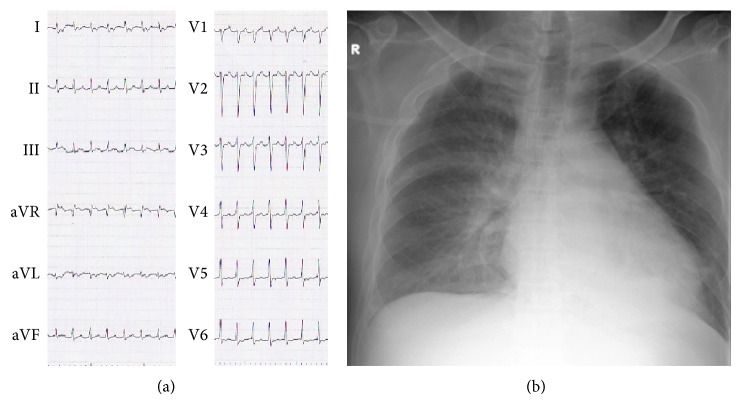
Twelve-lead electrocardiogram (ECG) (a) and chest X-ray on admission (b). ECG shows narrow QRS tachycardia suggesting paroxysmal supraventricular tachycardia. Chest radiography showed bilateral pulmonary congestion and cardiomegaly.

**Figure 2:**
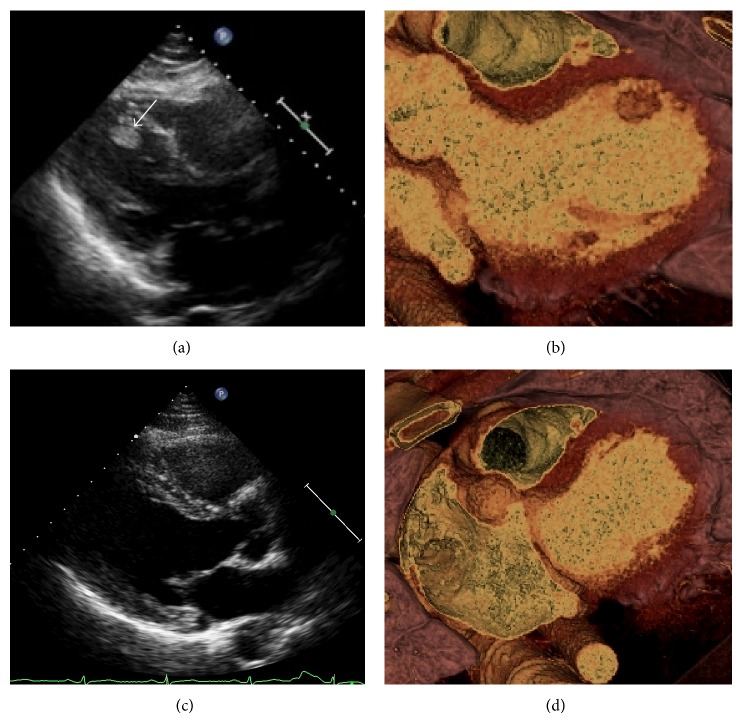
Imaging findings of a ball mass in the left ventricle (LV) on admission. This imaging revealed a ball mass (arrow) in the left ventricle (LV) detected by transthoracic echocardiography (TTE) (a) and contrast enhanced computed tomography (CT) (b). We confirmed that it had disappeared after anticoagulant therapy including rivaroxaban on the 14th day from TTE imaging (c) as well as CT imaging (d). Therefore, we diagnosed this ball mass as a thrombus.

**Figure 3:**
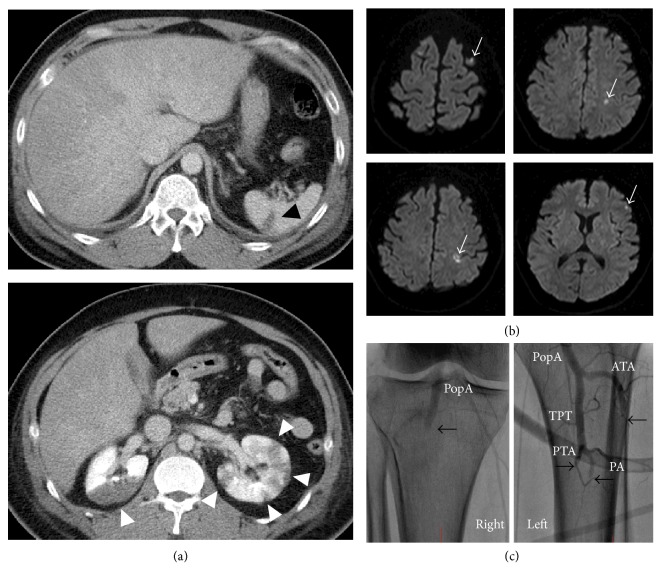
Imaging of systemic embolism. Axial views of contrast enhanced computed tomography on admission showed the splenic (black arrow head) and renal (white arrow head) embolisms (a). Also, head magnetic resonance imaging (diffusion weighted image) revealed cerebral infarctions (white arrow), which suggested they were due to shower cardiogenic embolism (b). Angiograms of bilateral below-the-knee arteries showed occlusions in the right popliteal artery (PopA), left anterior tibial artery (ATA), left peroneal artery (PA), and left posterior tibial artery (PTA) ((c); black arrows). TPT: tibioperoneal trunk.

Physical examination revealed a normal consciousness level and normal neurological findings. Blood pressure was 113/96 mmHg and heart rate was 132 beats per minute. Chest coarse crackle, jugular venous distention, and pitting edema on the lower extremities suggested heart failure. Both his feet were pale, which indicated the presence of acute limb ischemia. Blood tests revealed elevated levels of D-dimer (12.1 *μ*g/dL), brain natriuretic peptide (1,081 pg/mL), and white blood cells (22,200/*μ*L). Coagulation and fibrinolytic system abnormalities were not found—antithrombin activity was 131.1%, protein C and S activity were 63% and 89%, respectively, and anti-*β*2-glycoprotein 1 antibody and anticardiolipin antibody were negative. His renal function was not impaired (creatinine clearance estimated with Cockcroft-Gault formula: 108.8 mL/min). A 12-lead electrocardiogram (ECG) showed narrow QRS tachycardia with retrograde *P* wave, which we diagnosed as paroxysmal supraventricular tachycardia (PSVT) (Figure 1(a)). Chest radiography showed bilateral pulmonary congestion and cardiomegaly (cardiothoracic ratio 57%) (Figure 1(b)). Left ventricular wall motion was generalized hypokinetic (left ventricular ejection fraction [LVEF] was 15%) with distention of the inferior vena cava in transthoracic echocardiography (TTE). It also revealed an immobile ball mass was present in the left ventricle (LV) (20 × 10 mm) (Figure 2(a)) but not in the left atrium. We performed contrast enhanced computed tomography (CT), which revealed some contrast defects in the spleen and bilateral kidneys (Figure 3(a)) as well as in the LV (Figure 2(b)). In addition, head magnetic resonance imaging (MRI) (diffusion weighted image) showed small cerebral infarctions, possibly caused by shower embolism (Figure 3(b)).

Based on these results, we believed this patient suffered from heart failure secondary to tachycardia-induced cardiomyopathy and resultant systemic embolisms originating from the LV. Although there was a risk of further embolism, we decided to treat the arrhythmia to improve cardiac function and to prevent cardiac death. PSVT was restored to sinus rhythm by rapid injection of 20 mg of adenosine triphosphate (ATP) with no clinical signs and symptoms of additional thrombosis thereafter.

Catheter angiography revealed normal coronary arteries and occluded bilateral below-the-knee arteries (Figure 3(c)). For limb salvage, we performed endovascular therapies including thrombectomy with a Fogarty catheter in the right popliteal artery in association with an aspiration catheter for bilateral tibioperoneal vessels. All aspirated thrombi were found to be red and seemed to be fresh and not organized. The procedures were successful.

**Figure 4:**
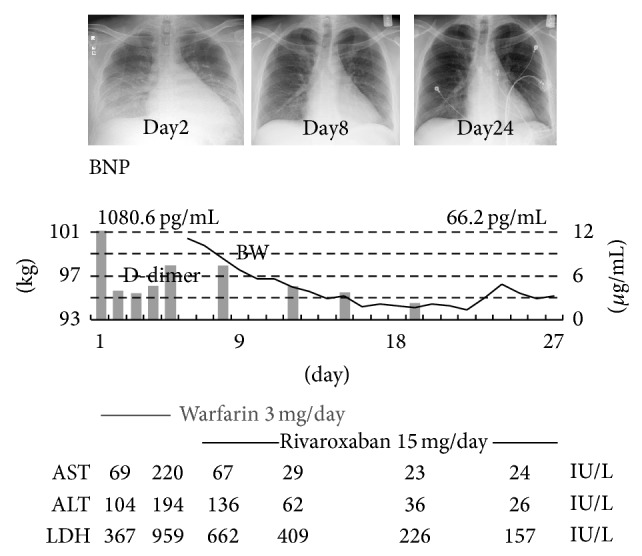
Clinical course. This figure demonstrates the course of acute heart failure and the changes of value of D-dimer and hepatic aminotransferase. Though hepatic function worsened by the fifth day, it improved gradually after switching anticoagulants.

The clinical course is shown in Figure 4. We started anticoagulation therapy with intravenous unfractionated heparin and warfarin (3 mg once daily) at the same time. The level of D-dimer declined by the following day but gradually elevated again slightly until the fifth day. On the same day, the prothrombin time-international normalized ratio (PT-INR) value rose to 4.78; therefore, anticoagulant therapies were discontinued to prevent further hemorrhaging. Moreover, as the levels of liver aminotransferases were also elevated, taking into account the hepatic effects of warfarin and since this patient emphatically refused intravenous drip injection treatments including heparin administration, we decided to switch from warfarin to the direct factor Xa (FXa) inhibitor rivaroxaban (15 mg once daily) after confirmation that the PT-INR value dropped to 1.85 on the seventh day. Although in Japan rivaroxaban for treating an LV thrombus is off label use, we used it after obtaining informed consent from this patient.

On the sixth day, transesophageal echocardiography (TEE) revealed the LV ball mass was the same size as on admission, but no thrombus was in the left atrial appendage (LAA). Surgical treatment was also being considered as a treatment option. However, before the final decision, the ball in the LV had vanished without clinical signs of cardiogenic embolism (Figures 2(c) and 2(d)) after 7 days of rivaroxaban, suggesting this ball mass was not a tumor but a thrombus.

His clinical course of heart failure was favorable and he also took verapamil orally for the prevention of PSVT attacks with careful attention to LV function. We performed cardiac electrophysiological examinations and radiofrequency catheter ablation of a lateral accessory pathway successfully. Since then, PSVT had not recurred. LVEF improved to 45% after the above treatments and he was discharged on the 27th day in a good medical condition under continuous anticoagulant treatment with the same dose of rivaroxaban.

## 3. Discussion

To the best of our knowledge, this is the first documented report that revealed LV thrombus resolution under short-term rivaroxaban treatment. There have been case reports of LV thrombus resolution with warfarin therapy [9, 10]. Similarly, some previously reported cases in recent years have shown the effectiveness of apixaban [6] and dabigatran [7, 11] for LAA and LV thrombus resolution. In 2013, a study reported that rivaroxaban was able to resolve LAA thrombus [8]. In this report, repeated TEE showed a markedly increased giant thrombus mass in the LAA under well-controlled VKA therapy for six weeks. After that the authors decided to switch the oral anticoagulation to rivaroxaban based on the increasing evidence that VKAs had a poor capability to resolve large intracardiac thrombi [8]. This new direct acting FXa inhibitor is reported to have the potential not only to prevent a thrombosis but also to resolve established thrombi by direct inhibition of free and thrombus-associated FXa [5]. Moreover, in a congestive heart failure rat model, rivaroxaban reduced platelet activation by attenuating the secondary phase of ADP-induced platelet aggregation [12]. Thus, rivaroxaban may constitute a useful approach to prevent thromboembolic complications and reduce platelet activation in congestive heart failure at the same time.

Furthermore, in the experimental study by Varin et al., it was concluded that rivaroxaban induced a modification of the fibrin network characterized by a looser plasma fibrin network with thicker fibers and larger pores. This resulted in a greater permeation of flow through the clots, rendering them more sensitive to fibrinolytic drugs [13]. Therefore the rivaroxaban itself may make thrombi easier to resolve.

As this patient was a young adult, we kept in mind a surgical option—LV thrombectomy. However, TTE on the 13th hospital day revealed the thrombus had disappeared. Since the period in this case from rivaroxaban administration to confirmation of disappearance of LV thrombus was shorter than that of past case reports (from 16 days to 6 weeks) [6–8, 11], it is possible that an additional embolism developed from the LV thrombus by the 13th day. We however did not see any additional symptoms and signs of embolism, although we did not perform contrast enhanced CT or head MRI during the hospitalization period. Another possible reason is that the antecedent warfarin and heparin treatment might have contributed to shortening of the period of LV thrombus resolution and a previous case report found that LAA thrombus was resolved by initial heparin therapy followed by 7 days of rivaroxaban treatment in two cases [14].

In this case, PSVT induced LV dysfunction leading to the LV thrombus formation. In a similar case [15], a patient suffered from atrial flutter and atrioventricular nodal reentrant tachycardia and severe heart failure caused by tachycardia-induced cardiomyopathy with thrombus in the LAA. Warfarin and catheter ablation were effective for their treatments [15]. We have to be mindful of the possibility that the sustained tachyarrhythmia can cause thrombus formation in any chambers of the heart and thromboembolism. Also, the management of PSVT with LV thrombus and tachycardia-induced heart failure was a point of discussion. Fortunately, this patient did not suffer from further thromboembolism just after abrupt termination of PSVT with ATP. Of course, both beta-blocker usage for gradual reduction of heart rate for prevention of thrombosis and a starting of anticoagulant treatment before termination of the PSVT are reasonable strategies. However, we did not use beta-blockers for PSVT because the congestive heart failure was more severe, and with a risk of further worsening, than the gradual decrease of heart rate. In this case, we believed that first his LV systolic motion was so impaired that rhythm control would not change the hemodynamics in the LV to a great extent. Moreover, the ball mass of thrombus looked immobile and we were not completely confident that the ball in the LV was thrombus at that time. Therefore, we decided to restore sinus rhythm for his recovery from heart failure as soon as possible. Based on the presumable location of the left accessory pathway from the electrocardiogram and presence of the LV thrombus, emergent catheter ablation was not considered.

The propriety of switching from warfarin to rivaroxaban should be discussed. When considering the relationship between reelevation of D-dimer and further elevation of AST and LDH on the fifth day retrospectively, it might be reasonable to conclude that thrombus resolution had already begun at that time by warfarin administration accompanying another embolism leading to AST and LDH elevation. However, we switched warfarin to rivaroxaban after considering the possibility of an allergic reaction to warfarin. Thus, we cannot completely deny the possibility that warfarin might have partly contributed to the resolution of the LV mass of thrombus.

Also, we cannot completely deny the possibility of additional distal embolism after rivaroxaban use, because we did not perform contrast enhanced CT or head MRI to confirm the absence of additional embolism after the disappearance of LV thrombus with TTE.

This case report showed the potential of rivaroxaban to resolve LV thrombus. More case reports and studies are warranted to prove the fibrinolytic effects of NOACs including rivaroxaban.
